# Two new species of the family Megalyridae (Hymenoptera) from China

**DOI:** 10.3897/zookeys.1043.65223

**Published:** 2021-06-10

**Authors:** Hua-yan Chen, Bo-jing Liuhe, Xiao Zhang

**Affiliations:** 1 State Key Laboratory of Biocontrol, School of Life Sciences / School of Ecology, Sun Yat-sen University, Guangzhou 510275, China State Key Laboratory of Biocontrol, School of Life Sciences / School of Ecology, Sun Yat-sen University Guangzhou China; 2 Kunming Daqiuyin Technology Co., Ltd, Kunming 650051, China Kunming Daqiuyin Technology Co., Ltd. Kunming China

**Keywords:** Megalyroidea, Oriental Region, parasitic wasps, taxonomy

## Abstract

Two new species of the small and rarely collected family Megalyridae are described from China: *Carminator daliensis* Chen & Liuhe, **sp. nov.** from Yunnan and *Ettchellsia hainanensis* Chen & Liuhe, **sp. nov.** from Hainan. A key to megalyrid species of China is provided. The biogeographical implication of the new taxa is discussed.

## Introduction

Megalyridae is a small family of parasitic wasps, which, as far as known, parasitize the larvae of wood-boring beetles (Coleoptera) and more rarely mud-nesting Sphecidae (Hymenoptera) (Shaw 1990a; Naumann 1987). So far, only 62 species have been described worldwide (Binoy et al. 2020; Mita and Shaw 2020). Although rarely collected, these wasps have nevertheless been reported from most of the major biogeographic regions worldwide, with the exception of the Nearctic (Vilhelmsen et al. 2010). Most megalyrid species occur in the tropics and subtropics of the Southern Hemisphere (Vilhelmsen et al. 2010; Binoy et al. 2020). While most Asian species of megalyrids have highly restricted distribution ranges (Mita and Konishi 2011), there is one notable exception, *Megalyra fasciipennis* Westwood, which occurs widely in Australia and Tasmania and has been reported as accidentally introduced into South Africa and India (Binoy et al. 2020). The biogeography of megalyrids has attracted increasing attention in recent years (Shaw 1990b; Vilhelmsen et al. 2010; Mita and Konishi 2011). Therefore, an intensive field survey of megalyrids is of interest towards better understanding the diversity and distributional patterns of these wasps. Previously, *Carminator cavus* Shaw from Taiwan and *Ettchellsia sinica* He from Yunnan were the only two megalyrids described from China (Shaw 1988; He 1991). In this study, we describe two additional new species of Megalyridae from southern China.

## Materials and methods

All specimens are deposited in the collections of the Museum of Biology, Sun Yat-sen University, Guangzhou, China (**SYSBM**). The morphological terms generally follow that of Shaw (1988, 1990b), and the nomenclature of the sculpture and texture of the integument follows Harris (1979). Images and measurements were made using a Nikon SMZ25 microscope with a Nikon DS-Ri 2 digital camera system. Images were post-processed with Abobe Photoshop CS6 Extended.

The following abbreviations are used:

**A1**–**A14** antennomere 1 to 14;

**OOL** shortest distance from the outer edge of a lateral ocellus to the compound eye;

**LOL** shortest distance between the inner edges of the lateral ocellus and the median ocellus;

**POL** distance between the inner edges of the lateral ocelli.

## Results

### Key to species of Megalyridae of China

**Table:** 

1	Posterior ocular orbits without groove (Fig. 1F); forewing with vein Rs between Rs + M and r-rs absent, apical segment of Rs absent or spectral (Fig. 2D)	**2**
–	Posterior ocular orbits with groove (Fig. 3E); forewing with vein Rs between Rs + M and r-rs tubular, apical segment of Rs tubular, arched towards stigma (Fig. 4D)	**3**
2	Median strip on frons distinctly deeper than longitudinal striae; ventral margin of mesepisternum swollen, more or less rounded	***Carminator cavus* Shaw**
–	Median strip on frons indistinct, not deeper than longitudinal striae (Fig. 1C); ventral margin of mesepisternum flat (Fig. 2B)	***Carminator daliensis* Chen & Liuhe, sp. nov.**
3	Frons irregularly rugose (Fig. 3C); clypeus largely punctate rugose; POL distinctly longer than OL (Fig. 3D); 5^th^ metasomal tergite smooth (Fig. 4C)	***Ettchellsia hainanensis* Chen & Liuhe, sp. nov.**
–	Frons coarsely reticulate; clypeus dorsal half finely punctate, ventral half smooth; POL shorter than OL; 5^th^ metasomal tergite finely transversely rugulose	***Ettchellsia sinica* He**

#### Carminator

Taxon classificationAnimaliaHymenopteraMegalyridae

Shaw, 1988

52BDE210-1E78-516A-AD9F-7089500B287F

Carminator Shaw 1988: 102; Shaw 1990b: 572; Mita et al. 2007: 202; Vilhelmsen et al. 2010: 663; Mita and Konishi 2011: 109. Type species: Carminator ater Shaw, 1988.

##### Diagnosis.

*Carminator* is diagnosed by the following morphological characters: shallow subantennal groove, mandible stout and with five teeth, head prognathous, wing venation reduced and pterostigma absent, fore tibia with a comb of stout spines, ovipositor strongly arched (Shaw 1990b).

##### Biology.

Little is known about the biology of *Carminator*, but these wasps have been suspected to be parasitoids of wood-boring larvae of Coleoptera (Mita and Konishi 2011).

##### Distribution.

Oriental, Australasian, and eastern Palaearctic regions.

#### Carminator daliensis

Taxon classificationAnimaliaHymenopteraMegalyridae

Chen & Liuhe sp. nov.

EB541979-2855-5034-B441-F72A20DFC032

http://zoobank.org/AB8033B3-FEB2-4309-AEC6-08E2572B4991

Figures 1 , 2

##### Diagnosis.

**Figure 1.**
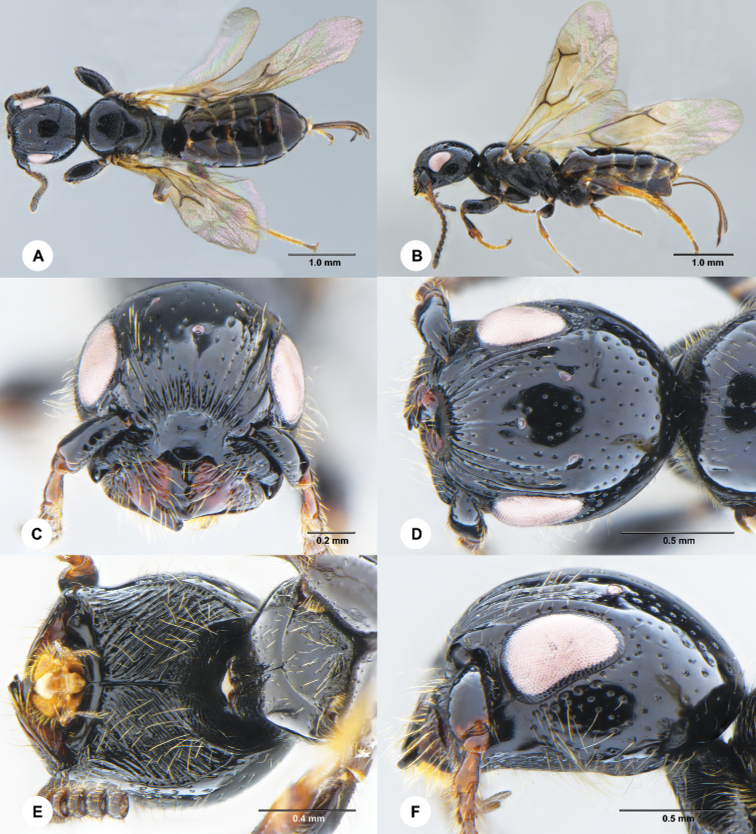
*Carminator daliensis* Chen & Liuhe, sp. nov., female, holotype (SCAU 3049430) **A** habitus, dorsal **B** habitus, lateral **C** head, frontal **D** head, dorsal **E** head and prothorax, ventral **F** head and pronotum, lateral.

**Figure 2.**
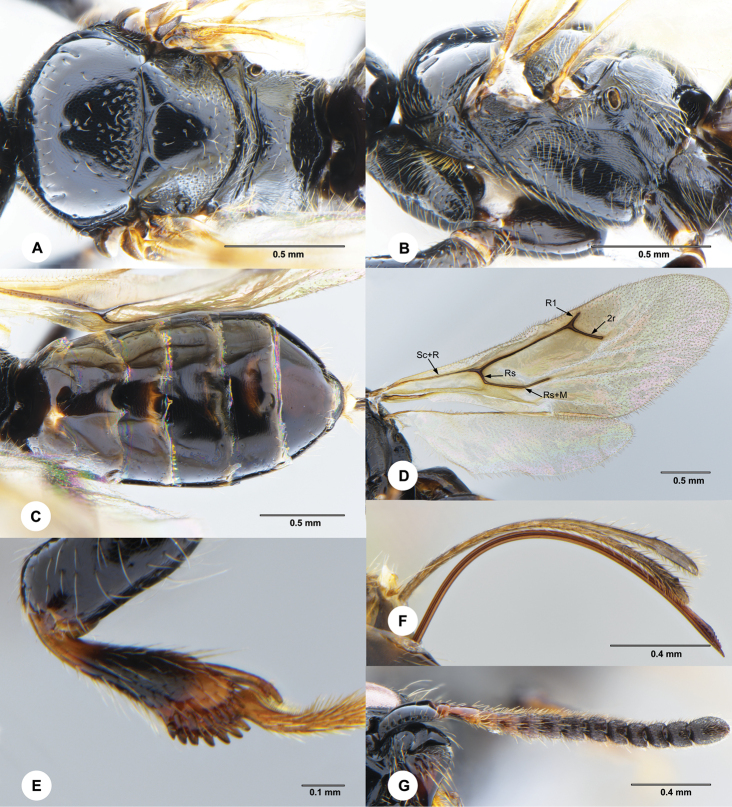
*Carminator daliensis* Chen & Liuhe, sp. nov., female, holotype (SCAU 3049430) **A** mesosoma, dorsal **B** mesosoma, lateral **C** metasoma, dorsal **D** wings **E** fore tibia, inner face **F** ovipositor and ovipositor sheath, lateral **G** antenna, dorsal.

Head longer than wide (Fig. 1D); frons entirely costate, median strip of frons shallow and smooth (Fig. 1C); postgena entirely obliquely striate (Fig. 1E); occipital ridge strongly arched (Fig. 1F); mandible with five blunt, subtriangular teeth (Fig. 1C); propleuron elongate, forming “neck” (Fig. 1E); prosternum without median groove (Fig. 1E); fore tibia with two rows of stout spines (nine + seven) arranged in a V shape (Fig. 2E); branching point between R1 and 2r of forewing not thickened (Fig. 2D).

##### Description.

**Female** (holotype). Body length 4.4 mm. *Color*. Body black; mandible reddish black; pedicel and first four flagellomeres dark brown, remainders of antenna black; legs black with tibiae dark brown to black and tarsus brown; wings tinged with brown and forewing veins dark brown; ovipositor sheath brown; ovipositor reddish brown.

***Head*** (Fig. 1C–F) 1.1× longer than wide in dorsal view, widely smooth with scattered punctures, punctures denser on gena and vertex; frons costate along lower margin, with about 12 longitudinal costae, punctures present among costae; median strip shallow and smooth; outer margin of frons without granulated area; posterior margin of frons slightly convex, higher than level of clypeus; punctures on gena and vertex denser; malar space below antennal insertion obliquely striate, with scattered punctures; posterior carina of subantennal groove present; lower margin of gena (as genal ridge in Mita and Konishi 2011) simple, not forming a blunt angle; postgena entirely obliquely striate; vertex wide, without a coronet behind lateral ocelli; occipital ridge arched; clypeus smooth, posterior margin rounded; a groove parallel to inner orbit of eye; eye with scattered short setae; ocelli forming large obtuse triangle; POL = 6.5; OL = 4.9; OOL = 3.2; mandible with five blunt subtriangular teeth, basal part swollen, outer margin flat; antenna (Fig. 2G) with A1–A3 weakly flattened, A4–A14 strongly flattened, covered with numerous short to long setae; length/width ratio of antennal segments: 6.4:2.0, 1.3:1.3, 3.0:1.5, 1.8:1.9, 2.0:2.1, 2.2:2.5, 2.2:2.5, 2.2:2.5, 2.2:2.4, 2.1:2.4, 1.8:2.3, 2.1:2.5, 2.2:2.6, and 4.4:2.7.

***Mesosoma*** (Figs 1E, F, 2A, B). Pronotum shagreened to rugulose; propleuron dorsally shagreened, ventrally coriaceous, elongate, forming “neck”; median groove of prosternum absent; setae on propleuron and prosternum dense; mesoscutum largely smooth with sparse, small punctures anteriorly and laterally, coriaceous to finely punctate rugulose posteriorly, median mesoscutal sulcus weakly developed and only present anteriorly; admedian lines absent, parapsides short and present anterolaterally; mesoscutum convex, 0.65× as long as wide; mesoscutellum 0.57× as long as wide, coriaceous with sparse puntures medially, finely and desenly punctate laterally; anterior margin of axillae broadly separated; mesopleuron rugulose dorsally, largely shagreened or coriaceous ventrally; epicnemial sulcus obscure; ventral margin of episternum forming blunt angle; episternal scrobe with a weak depression; pleural sulcus complete; metanotum narrow, strongly shagreened, without punctures; metapleuron swollen anteriorly, covered with long setae; propodeum with posterior propodeal carina distinct, area anterior to posterior propodeal carina largely shagreened with sparse small punctures medially, area posterior to posterior propodeal carina smooth.

***Legs*.** Fore tibia flat, with two rows of stout spines arranged in a V shape (Fig. 2E); fore tarsomeres with ratios: 7.3:2.6:2.1:1.6:3.8; hind tarsomeres with ratio: 5.4:1.9:1.4:1.0:3.7.

***Wings*** (Fig. 2D). Forewing with vein Sc + R branching into veins R and Rs; R1/2r = 0.4; branching point between veins R1 and 2r not thickened; Rs/(Rs + M) = 0.18.

***Metasoma*** (Fig. 2C, F) subcylindrical, widest at metasomal segment 4; terga and sterna faintly shagreened to smooth; tergite 1 0.43 × as long as wide; ovipositor sheath 0.9× as long as ovipositor; setae on ovipositor sheath longer than diameter of ovipositor; apex of ovipositor sharp.

**Variation.** The body length of the paratype female is 4.2 mm, and other characters are similar to the holotype.

**Male.** Unknown.

##### Etymology.

The specific epithet refers to the locality (Dali) where the type specimens were collected. It should be treated as a noun in apposition.

##### Material examined.

***Holotype*,** female, China: Yunnan, Dali, Yunlong County, 3063 m a.s.l., forest, 21°51'23"N, 99°14'10"E, 12–27.ix.2020, Malaise trap, SCAU 3049430 (SYSBM). ***Paratype***: 1 female, same collecting data as holotype, SCAU 3049431 (SYSBM).

##### Distribution.

Oriental region, China, Yunnan Province.

#### Ettchellsia

Taxon classificationAnimaliaHymenopteraMegalyridae

Cameron, 1909

F51700BD-211D-501A-9A1A-FAEEEDFFA825

Ettchellsia Cameron 1909: 208; Baltazar 1961: 219; He 1991: 475; Mita and Shaw 2012: 101. Type species: Ettchellsia piliceps Cameron, 1909 (by monotypy).

##### Diagnosis.

Posterior ocular orbits with groove and carina present; posterior border of mesopleuron smooth, without a row of foveae; propodeum with unique pattern of longitudinal carinae; forewing fuscous or with fuscous banding pattern; forewing with vein Rs between Rs + M and r-rs tubular for at least a short distance, apical segment of Rs tubular, arched towards stigma, M+ Cu and distal segments of Cu absent or at most spectral; Hind tibia rugose and with erect setae. Additional diagnostic characters for the genus were provided by Shaw (1990b), Vilhelmsen et al. (2010), and Mita and Shaw (2012).

##### Biology.

No biological data for *Ettchellsia* species are available; however, these wasps have long been suspected to be idiobiont ectoparasitoids that attack beetle larvae (Shaw 1990b).

##### Distribution.

Oriental region.

#### Ettchellsia hainanensis

Taxon classificationAnimaliaHymenopteraMegalyridae

Chen & Liuhe sp. nov.

D9266F91-7C16-58A3-97B3-4B10B018AB0E

http://zoobank.org/0DE06E91-FC9A-43C8-88FF-36C2FCA1CCCF

Figures 3 , 4

##### Diagnosis.

**Figure 3.**
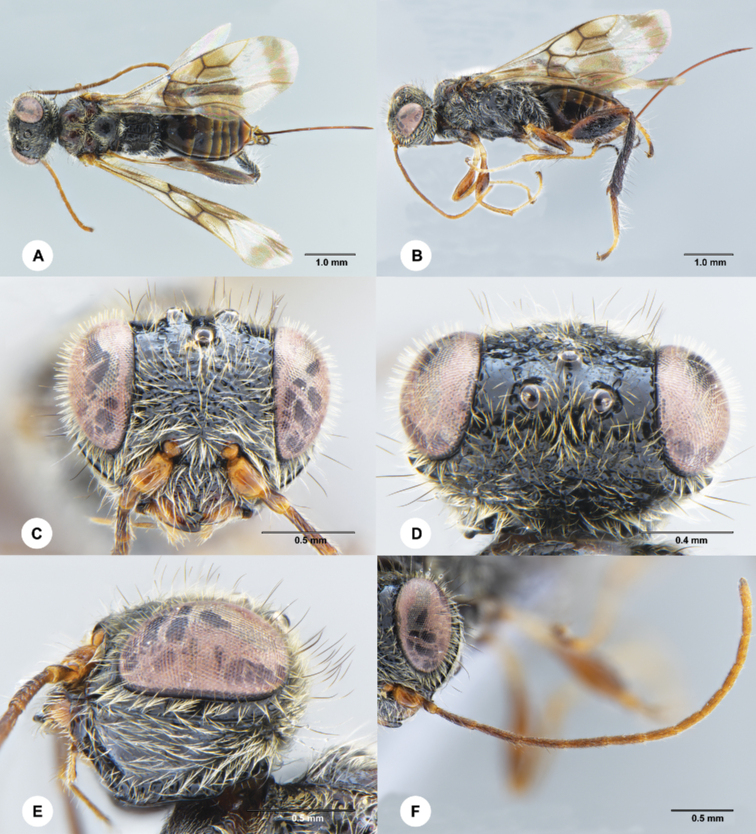
*Ettchellsia hainanensis* Chen & Liuhe, sp. nov., female, holotype (SCAU 3049429) **A** habitus, dorsal **B** habitus, lateral **C** head, frontal **D** head, dorsal **E** head, lateral **F** antenna.

**Figure 4.**
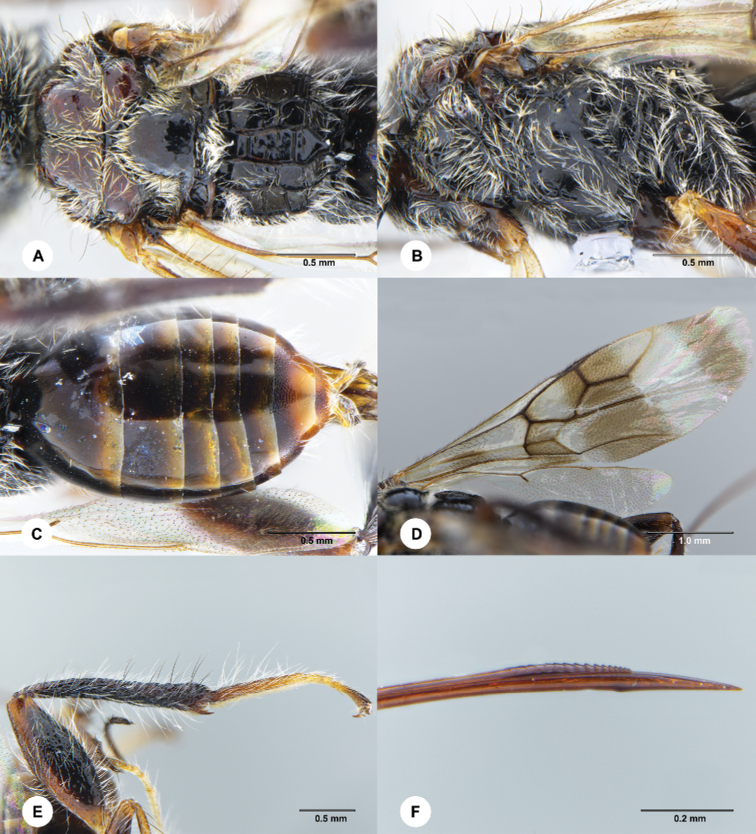
*Ettchellsia hainanensis* Chen & Liuhe, sp. nov., female, holotype (SCAU 3049429) **A** mesosoma, dorsal **B** mesosoma, lateral **C** metasoma, dorsal **D** wings **E** hind leg, lateral **F** apical ovipositor, lateral.

Frons irregularly rugose (Fig. 3C); clypeus largely punctate rugose (Fig. 3C); vertex posterior to lateral ocellus smooth anteriorly and reticulate-rugose posteriorly (Fig. 3D); gena smooth (Fig. 3E); metanotum puncate and setose medially (Fig. 4A); median propodeal region not narrowed (Fig. 4A); metasoma (Fig. 4C) smooth except tergite 6 largely coriaceous.

##### Description.

**Female** (holotype). Body length 4.9 mm. *Color*. Head black, mesosoma largely black except mesoscutum and axill reddish brown and tegula brown, metasoma mainly black with posterior margin of terga dark brown; mandible dark brown with teeth darker; antenna brown to dark brown with apical flagellomeres paler; trochanters and tarsi of fore and mid legs pale yellow, remainders of the legs brown to dark brown; trochanter and tarsus of hind leg brown, femur dark brown to black, coxa and tibia black; dorsal surface of hind tibia with both white and black long setae; basitarsus with white long setae; forewing with four transverse dark brown bands; ovipositor sheath black; ovipositor reddish brown.

***Head*** (Fig. 3C–E) 1.5× wider than long in dorsal view, covered with long black erect setae and relatively short decumbent white setae; frons irregularly rugose; vertex convex, with ocellar triangle smooth, except by a median row of longitudinal punctures, area between ocelli and eyes smooth with a row of longitudinal punctures arising from lateral ocellus and parallel to orbit; vertex posterior to lateral ocellus smooth anteriorly and reticulate-rugose posteriorly; POL = 4.7; OL = 3.4; OOL = 4.0; eye margined posteriorly by a groove and a single postocular oribital carina, the groove smooth anteriorly and foveate posteriorly; gena smooth; occipital carina foveate; clypeus largely punctate rugose, apical margin slightly incise medially; mandible with 3 teeth; antenna filiform, A3–A6^th^ flagellomeres subequal and the longest, remainder flagellomeres becoming shorter.

***Mesosoma*** (Fig. 4A, B) covered with short decumbent white setae; scattered, long, erect, black setae present on mesonotum; mesoscutum humped, anterior surface smooth, dorsal surface largely smooth with fine punctures, median mesoscutal sulcus present and foveate, lateral carina of anterior surface present; axilla and mesoscutellum largely smooth with fine punctures; metanotum puncate and setose medially; propodeum with pairs of median, submedian, and lateral longitudinal carinae; median propodeal region not narrowed, with four complete and two incomplete transverse carinae, posterior margin producing dorsally; submedian region with two complete transverse carinae; lateral region with four complete transverse carinae.

***Legs*** (Figs 3B, 4E). Hind femur punctate and rugose medially; hind tibia longitudinally rugose; hind tibia covered with both white and black long erect setae, longer than the width of the hind tibia; basitarsus covered with white long erect setae, longer than the width of the hind tibia.

***Wings*** (Fig. 4D). Forewing with vein M 1.9× basal part of vein RS; erect setae on vein C 0.5× those on vein Sc+R and A.

***Metasoma*** (Fig. 4C) smooth except tergite 6 largely coriaceous; ovipositor 1.76× mesosoma length, apex with small teeth and single knob.

**Variation.** The body length of the paratype female 5.0 mm; antenna dark brown to black; mesoscutellum reddish brown; median propodeal region with seven complete transverse carinae; other characters similar to the holotype.

**Male.** Unknown.

##### Etymology.

The specific epithet refers to Hainan Island, where the type locality is located. It is treated as a noun in apposition.

##### Material examined.

***Holotype*,** female, China: Hainan, Qiongzhong, Mount Limushan, 19°10.771'N, 109°46.225'E, 20–22.vii.2020, forest, sweep, Huayan Chen, SCAU 3049429 (deposited in SYSBM). ***Paratype***: 1 female, China: Hainan, Qiongzhong, Mount Limushan, 19°10'23.28"N, 109°46'40.79"E, 30.xi–31.xii.2020, forest, Malaise trap, Longlong Chen, SCAU 3042295 (SYSBM).

##### Distribution.

Oriental region, China, Hainan Province.

##### Remarks.

*Ettchellsia hainanensis* is most similar to *E. sinica* He, which was previously described based on a single female from Yunnan of China, but *E. hainanensis* can be distinguished from *E. sinica* by the following characters: frons irregularly rugose (Fig. 3C); clypeus largely punctate rugose (Fig. 3C); POL distinctly longer than OL (Fig. 3D); metasomal tergite 5 smooth (Fig. 4C). We contacted the curators of the Hymenoptera collection of Zhejiang University (previously known as Zhejiang Agricultural University) where the holotype of *E. sinica* claimed to be deposited, but they failed to find the type. If the holotype were confirmed to be lost, a neotype of the species may need to be designated based on a specimen collected from the type locality in the future.

## Dicussion

Mita and Shaw (2012) have suggested that species diversity of megalyrids from the Southeast Asia is still undersampled and intensive study is required. Additional discoveries from this region would help us better understand the biogeography and eveolutionary history of Megalyridae.

Species of *Carminator* mainly occur in Southeast Asia. Morphologically, *C. daliensis* is most similar to *C. affinis* Shaw, but the former can be distinguished by its smaller size and that frons entirely costate (only laterally costate in *C. affinis*) and prosternum without median groove (prosternum with median groove in *C. affinis*). *Carminator daliensis* is also similar to *C. ater* in having frons entirely costate, but the vertex is largely smooth with sparse punctures and the dorsal carina of subantennal groove is present. Geographically, *C. daliensis* is close to *C. affinis* from Malaysia and *C. ater* from Thailand (Shaw 1988). According to Mita and Konishi (2011), *Carminator* is a monophyletic taxon, with *C. affinis* as the sister group to the rest of the genus. So, the discovery of *C. daliensis* and its morphological similarity to *C. affinis* might serve as additional evidence that the common ancestor of the extant *Carminator* species was probably present in the Oriental–Australian transition zone and subsequently species dispersed northward as far as Japan (Mita and Konishi 2011).

So far, including the *E. hainanensis* described here, *Ettchellsia* species have mainly been found in the Indomalayan region (Mita and Shaw 2012). Unidentified species of *Ettchellsia* were reported from Taiwan (Shaw 1990b; Vilhelmsen et al. 2010) where the northernmost record of the genus is located. Further biogeographical and phylogenetic analyses of *Ettchellsia* will be desired in the future when additional new species and distributional records are found.

## Supplementary Material

XML Treatment for Carminator

XML Treatment for Carminator daliensis

XML Treatment for Ettchellsia

XML Treatment for Ettchellsia hainanensis
